# Dysregulation of sphingolipid metabolism in pain

**DOI:** 10.3389/fphar.2024.1337150

**Published:** 2024-03-08

**Authors:** Jianfeng Wang, Guangda Zheng, Linfeng Wang, Linghan Meng, Juanxia Ren, Lu Shang, Dongtao Li, Yanju Bao

**Affiliations:** ^1^ Department of Oncology, Guang’anmen Hospital, China Academy of Chinese Medical Sciences, Beijing, China; ^2^ Liaoning University of Traditional Chinese Medicine, Shenyang, Liaoning Province, China

**Keywords:** sphingolipid, sphingolipid metabolism, S1P, neuropathic pain, cancer-related pain

## Abstract

Pain is a clinical condition that is currently of great concern and is often caused by tissue or nerve damage or occurs as a concomitant symptom of a variety of diseases such as cancer. Severe pain seriously affects the functional status of the body. However, existing pain management programs are not fully satisfactory. Therefore, there is a need to delve deeper into the pathological mechanisms underlying pain generation and to find new targets for drug therapy. Sphingolipids (SLs), as a major component of the bilayer structure of eukaryotic cell membranes, also have powerful signal transduction functions. Sphingolipids are abundant, and their intracellular metabolism constitutes a huge network. Sphingolipids and their various metabolites play significant roles in cell proliferation, differentiation, apoptosis, etc., and have powerful biological activities. The molecules related to sphingolipid metabolism, mainly the core molecule ceramide and the downstream metabolism molecule sphingosine-1-phosphate (S1P), are involved in the specific mechanisms of neurological disorders as well as the onset and progression of various types of pain, and are closely related to a variety of pain-related diseases. Therefore, sphingolipid metabolism can be the focus of research on pain regulation and provide new drug targets and ideas for pain.

## 1 Introduction

The IASP describes pain as an unpleasant sensory and emotional experience associated with or similar to actual or potential tissue damage (Zou et al., 2021). Persistent peripheral stimulation or tissue damage can lead to central and peripheral neuropathic changes that result in chronic pain (Treede et al., 2019). Several diseases, including cancer, diabetic neuropathy, and multiple sclerosis, involve changes in the nervous system microenvironment, leading to changes in neuronal plasticity with nociceptive hypersensitivity, and the development of pain as a long-term concomitant symptom, which can seriously affect the quality of life. However, existing pain management programs are not fully satisfactory. Therefore, there is a need to dig deeper into the pathological mechanisms of pain generation and to find new targets for drug therapy.

All eukaryotic cells are surrounded by membranes composed of lipid bilayers, and sphingolipids, as major players in the composition of the cellular membrane skeleton, play an important role in the maintenance of normal cellular activities, signaling, and intercellular connections. Sphingolipids consist of sphingoid longchain base, fatty acids, and head groups as constitutive elements. And their intracellular synthesis and metabolic pathways constitute a huge network that is regulated by a variety of enzymes, with great complexity and diversity, among which ceramides are the key pivot of the SLs biosynthetic pathway (Lahiri and Futerman, 2007). A large number of specific sphingolipids have been shown to play key regulatory roles in intracellular signaling with potent bioactivities that produce important cell-based effects. Sphingolipids and cholesterol dynamically aggregate in cell membranes to form lipid rafts, which are involved in attachment of proteins and signaling (Simons and Ikonen, 1997). These lipid rafts are involved in many cellular events such as proliferation, differentiation, motility, growth, senescence, and apoptosis, and further influence the course and development of diseases including cancer metastasis, autoimmune diseases, inflammatory diseases, and neurological disorders (Lahiri and Futerman, 2007).

Sphingolipids are widely found in the central nervous system and are important players in neurological disorders (Hannun and Obeid, 2017). Stabilization of sphingolipid metabolism, especially the ceramide and S1P metabolic pathways, is essential for the maintenance of nervous system function (Salvemini et al., 2013). Defects in sphingolipid metabolism, on the other hand, can lead to a variety of neurodegenerative pathologies. For example, accumulation of amyloid-β (Aβ) stimulates hydrolysis of sphingolipids, increases ceramide levels, and contributes to the development of Alzheimer’s disease (AD) and neurodegeneration (van Echten-Deckert and Walter, 2012; Alaamery et al., 2020). In addition, sphingolipid metabolism can induce the expression of multiple inflammatory mediators by interfering with glial cell phenotypes, leading to a variety of neuroinflammatory disorders, including pain (Lee et al., 2020). It has been shown that chronic pain is associated with abnormal serum lipid markers, and the highest correlation between sphingolipids and pain has been noted by LC/MS analysis (Gonzalez et al., 2022).

Therefore, this review focuses on an overview of the specific mechanisms of sphingolipids and sphingolipid metabolism in the development of pain as well as a discussion of sphingolipids as potential targets for pain management.

## 2 Sphingolipids and sphingolipid metabolism

**FIGURE 1:**
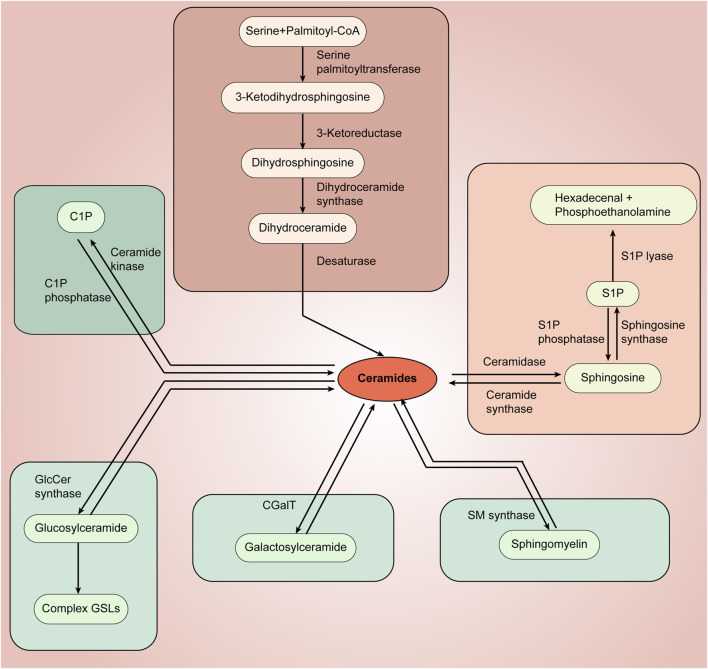
Sphingolipids and sphingolipid metabolism pathway.

**FIGURE 2:**
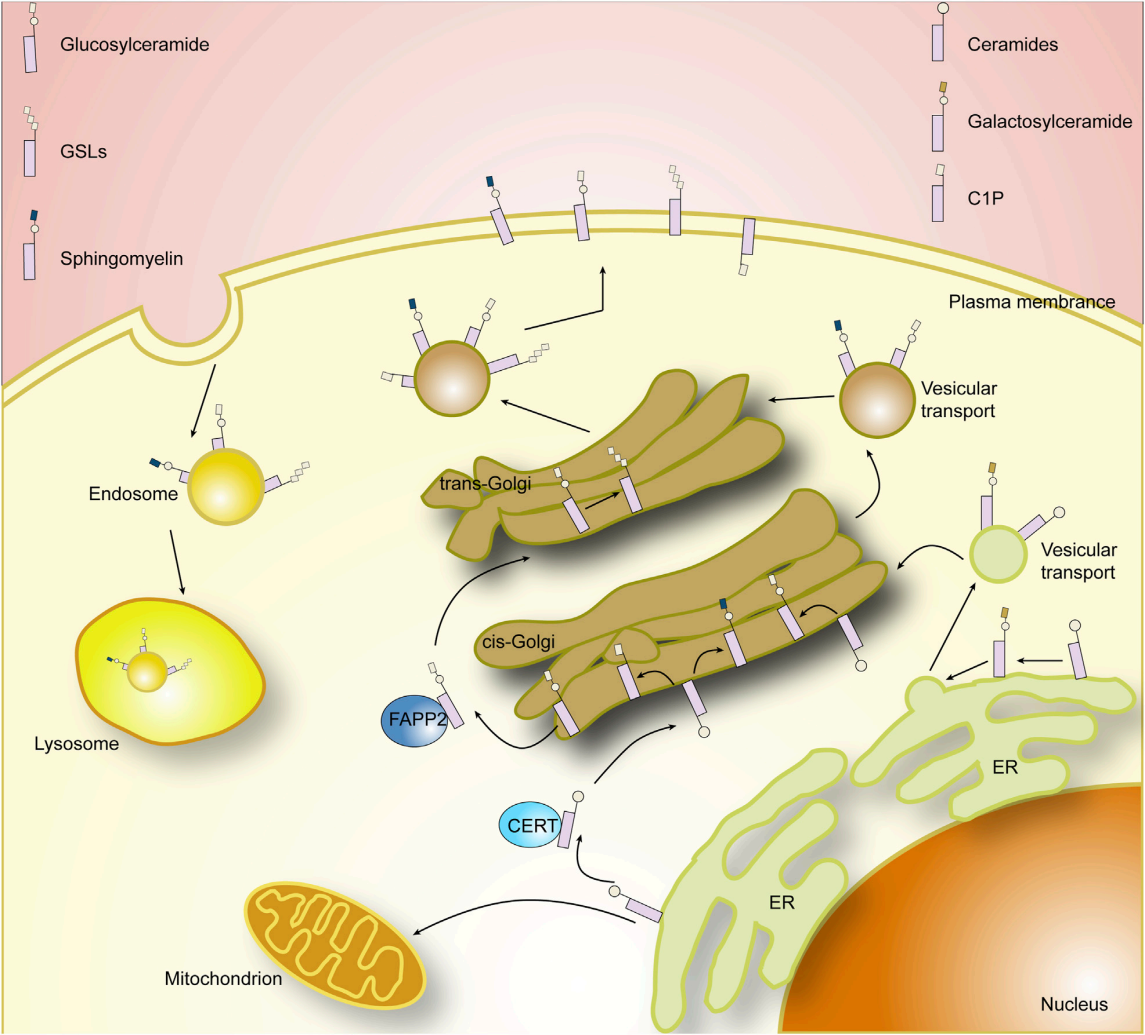
Cellar sphingolipid metabolism.

The membrane skeleton of eukaryotic cells is composed of a lipid bilayer, and the major components of the lipid bilayer that have been identified include glycerolipids, sphingolipids, and sterols. Sphingolipids are a class of lipids prevalent in eukaryotic cells and are important participants in cellular activities. The production and metabolism of sphingolipids form a complex network of interactions, in which each component plays a role in the formation of cell membranes, the regulation of intracellular signaling, and the regulation of intercellular activities (Bartke and Hannun, 2009). Sphingolipids were first discovered in 1876 by J. L. W. Thudichum, and they are a class of lipids defined by the synthesis of sphingoid longchain base (mainly sphingosine, phytosphingosine, and dihydrosphingosine) from the endoplasmic reticulum as a backbone (Merrill et al., 1997). To the base backbone can be further attached fatty acids via amide bonds and a polar head group (ranging from simple hydrogens to highly complex glucose) at the hydroxyl group, which constitutes the three basic building blocks of sphingolipids, the three main elements and their respective diversities that define the complexity of the sphingolipid family (Futerman and Riezman, 2005). Depending on their head groups, sphingolipids can be divided into three categories: ceramides, phosphosphingolipids (PSLs), and glycosphingolipids (GSLs), and in mammals alone, the head groups contain hundreds of variants (Lahiri and Futerman, 2007) (Figures 1, 2).

The synthesis of sphingolipids initially occurs in the cytoplasmic leaflets of the endoplasmic reticulum, where 3-ketosphingosine is synthesized from serine and palmitoyl coenzyme A at serine palmitoyltransferase (SPT) (Hanada, 2003). The main genes found to affect SPT are SPT1, SPT2, and SPT3 (Hornemann et al., 2006). Mutations in the genes encoding SPT1 and SPT2 have been demonstrated to be associated with hereditary sensory and autonomic neuropathies associated (Bejaoui et al., 2001; Dawkins et al., 2001). Subsequently 3-ketosphingosine is reduced to sphingosine by 3-ketoarginine reductase, followed by dihydroceramide synthetase, which then acylates sphingosine (SPH) and adds different fatty acyl chains to the backbone to form dihydroceramides. Six different genetically encoded dihydroceramide synthetases have been identified in mammals, and there are marked differences in the expression of different dihydroceramide synthetases in different tissues (Jeon et al., 2001; Hannun and Obeid, 2017). Dihydroceramide, in turn, is immediately followed by the generation of ceramide by dihydroceramide desaturase, which is the central hub of sphingolipid biosynthesis and a precursor of complex sphingolipids (Michel and van Echten-Deckert, 1997). Notably, phytoceramides are intermediate reaction products in the conversion of dihydroceramide phase ceramides (Christopher et al., 2010).

The next critical step in sphingolipid synthesis is the further modification of ceramides to form sphingolipids and glycosphingolipid, which occurs mainly in the endoplasmic reticulum and Golgi apparatus, where galactosylceramide synthesis occurs mainly in the endoplasmic reticulum and is catalyzed by ceramide galactosyltransferase (CGT/CGalT) (Stahl et al., 2004). Glucosylceramide and sphingomyelin (SM), on the other hand, occur mainly in the Golgi apparatus and require the transfer of ceramide from the endoplasmic reticulum to the Golgi apparatus. Ceramides, as membrane-bound molecules with very low solubility in water, are transported in two main ways. One is by binding to the protein ceramide transfer protein (CERT), which contains domains that can reside in the endoplasmic reticulum and recognize the Golgi membrane, respectively, and a hydrophobic domain that binds to ceramide to transport it (Hanada et al., 2007; Kumagai et al., 2007). The other mode of transport is via vesicles, a process that relies on COPII complex (Watson and Stephens, 2005). After being successfully transferred to the Golgi, ceramides can be synthesized into glucosylceramide in the presence of the glucosylceramide synthase (GCS/GlcCer). The newly produced glucosylceramide can be recognized and transported by FAPP2, and the glucosylceramide will go on to synthesize complex glycosphingolipid in downstream reactions (Jeckel et al., 1992). Alternatively, sphingomyelin are generated by transferring phosphocholine from phosphatidylcholine to ceramide in the presence of sphingomyelin synthase (SMS) (Huitema et al., 2003). The newly generated sphingomyelin and glucosylceramides will be transported to the plasma membrane via vesicles. Interestingly, in addition to being converted to more complex sphingolipids by CGT, GCS, and SMS, ceramides can be phosphorylated at the Golgi or at the plasma membrane to generate ceramide-1-phosphate (C1P), and the enzymes involved in this process are primarily ceramide kinases. C1P generated in the Golgi can be selectively translocated to the plasma membrane via a non-vesicular mechanism by binding to a specific transporter, human C1P transfer protein (CPTP) (Zhang et al., 2021). Thus, CPTP also affects specific SLs levels and thus regulates cellular SLs homeostasis.

In addition, it is worth noting that lipids, due to their hydrophobic nature, are not easily excreted and can cause intracellular accumulation, and thus the breakdown and destruction of sphingolipids is essential for the maintenance of intracellular homeostasis. Each enzyme that mediates the production of a specific sphingolipid is accompanied by an enzyme that breaks down its products, allowing a coordinated balance between intracellular sphingolipid production and metabolism without excessive accumulation of substrates. The complex sphingolipids are removed from the plasma membrane and are progressively degraded to ceramides by the action of enzymes, the respective components being recycled in the cell (Quinville et al., 2021). Among them, sphingolipids are hydrolyzed by sphingomyelinases to produce ceramides and free phosphocholine, whereas mammalian sphingomyelinases comprise three classes of neutral, acidic, and alkaline sphingomyelinases depending on PH and partitioning (Bartke and Hannun, 2009). Ceramides are then metabolized by ceramidase (CDSs) to form SPH, and CDSs can likewise be divided into neutral, acidic, and basic categories (Mao, 2008; Jozefczuk et al., 2020). SPH can be used to recycle into the SLs pathway or be phosphorylated by the SPH kinases to form S1P (Johnson et al., 2003). Similarly, S1P in turn can remove a phosphate group in the presence of S1P phosphatase to form SPH, or be further metabolized by the S1P cleavage enzyme catabolism (Bandhuvula and Saba, 2007). It has been shown that a variety of sphingomyelinases and ceramidase are present in the plasma membrane, Golgi, lysosomes, mitochondria, endoplasmic reticulum, and nucleus, respectively (Hannun and Obeid, 2017).

## 3 The ceramide-SPH-S1P axis in sphingolipid metabolism

**FIGURE 3:**
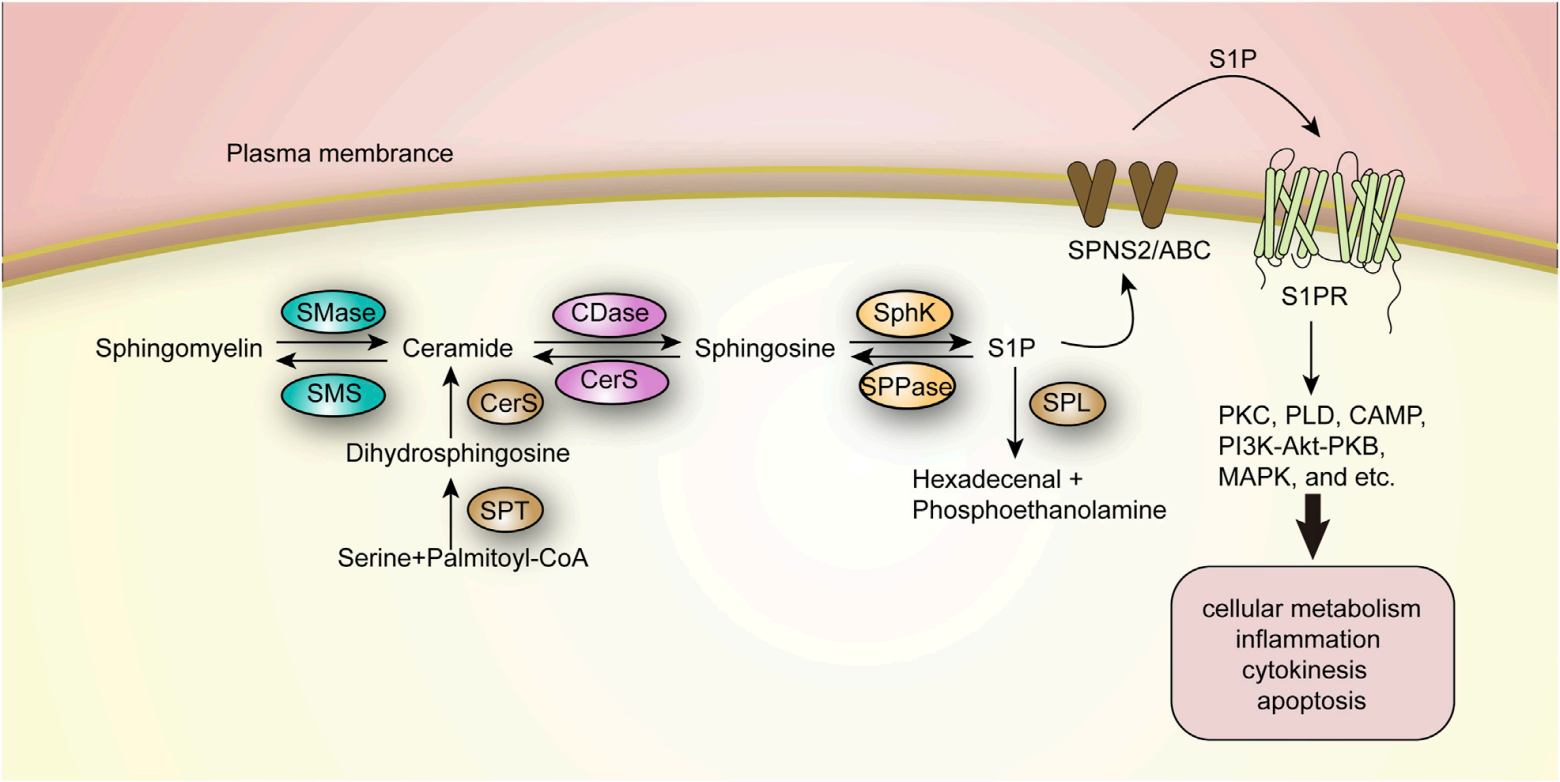
The Ceramide-SPH-S1P axis in sphingolipid metabolism.

Sphingolipids, as biologically active lipid molecules, serve important regulatory roles in physiopathologic processes such as apoptosis, autophagy, intercellular communication, and inflammatory and stress responses. Ceramides are precursor compounds for the synthesis of complex sphingolipids, and throughout sphingolipid metabolism, all complex sphingolipids are enzymatically broken down back into ceramides. Sphingomyelins are hydrolyzed by sphingomyelinases (SMase) to produce ceramides and free phosphocholine, and glycosphingolipids are also broken down to ceramides by their specific hydrolyzing enzymes, galactosylceramides and glucose ceramides, which are further broken down to ceramides by galactolactonase and β-glucosidase (Hannun and Obeid, 2008; Castro et al., 2014). As the basic skeleton of the complex sphingolipids that it constitutes and an important intracellular biologically active substance, ceramides is a central link and synthetic hub in the complex sphingolipid metabolic network. Notably, in the entire ceramide-centered sphingolipid metabolic network, the ceramide-SPH-S1P axis is a hotspot of research and an important target related to a variety of diseases (Figure 3).

Specifically, ceramide is deacylated by ceramidase and catabolized to SPH, which is either phosphorylated to S1P by SPH kinase (SphK) or recycled in the metabolic cycle to resynthesize ceramide. This process can occur in the mitochondria, plasma membrane, nucleus, and lysosomes (Quinville et al., 2021). This metabolic pathway is reversible, whereas S1P can be further degraded by sphingosine-1-phosphate lyase (SPL) to produce hexadecenal and phosphoethanolamine, completing the final step of sphingolipid catabolism, which is irreversible (Maceyka and Spiegel, 2014). And five ceramidases have been identified, divided into three categories: acidic, neutral and basic, encoded by different genes and mainly distributed in different compartments; for example, basic ceramidases are mainly found in the endoplasmic reticulum and Golgi. The SPH kinases involved in the formation of S1P are mainly SphK1 and SphK2, both of which are present as cytosolic enzymes. SphK1 is phosphorylated mainly by ERK2 kinase to achieve translocation from the cytosol to the plasma membrane, which then facilitates S1P production and extracellular release (Jeon et al., 2001; Pitson et al., 2005).

Ceramides were earlier shown to be associated with cellular stress response and apoptosis. Stimulation by deleterious factors induces sphingomyelinase to produce ceramides in large amounts, leading to the formation of ceramide-rich membrane structural domains in the cell (Schenck et al., 2007). Ceramide-rich membrane structural domains can mediate the activation of a variety of receptor molecules, such as mediating the aggregation of CD95, initiating and inducing apoptosis (Jeon et al., 2001). In vascular tissues, ceramide production and aggregation are involved in NO signaling and promotion of inflammation, whereas elevated blood ceramides are associated with the progression of several cardiovascular diseases (Zietzer et al., 2022). In addition, multiple studies have demonstrated the relevance of abnormalities in ceramide and its related enzymes to metabolic disorders, neurodegeneration, and cancer progression (Raichur, 2020; Zhang et al., 2023).

S1P is likewise a potent biologically active intracellular lipid second messenger that regulates a variety of biological processes by binding to intracellular targets or specifically to cell surface G protein-coupled receptors (S1PR1–S1PR5) (Prager et al., 2015). S1P shall be transported across the plasma membrane to the extracellular compartment via the Spinster 2 (SPNS2) transporter protein or a non-specific ABC transporter protein, and subsequently binds to S1PR in the extracellular compartment (Squillace et al., 2020). By binding to the receptor, S1P further activates downstream signaling cascades such as protein kinase C (PKC), phospholipase D (PLD), cyclic adenosine monophosphate (CAMP), PI3K-Akt-PKB (protein kinase B), and mitogen-activated protein kinase (MAPK), and thus participates in a wide range of events including cellular metabolism, inflammation, and cytokinesis and apoptosis, and the abnormalities in their catabolism have been shown to be importantly linked to several aspects including autoimmunity and inflammation, tumor microenvironment, tumor metastasis and vascular endothelial stability (Pitson et al., 2005; Levkau, 2013; Czubowicz et al., 2019).

## 4 Mechanisms of pain onset

Whether acute or chronic, noxious stimuli originating from peripheral nerves are transmitted through peripheral nerves to the spinal cord, which in turn uploads to different regions of the central nervous system to produce pain (Dinakar and Stillman, 2016). First, the noxious stimulus is converted into an action potential at the peripheral nociceptors, lowering the sensory threshold and leading to sensitization of the nociceptors and generation of nerve impulses (Garland, 2012). Such peripheral sensitization is initiated by peripheral bradykinin, histamine, and other mediators that are secreted at the site of injury and consist of a variety of chemicals called the “inflammatory broth” that stimulate the injurious receptors (Dinakar and Stillman, 2016). Subsequent changes in electrical potentials along primary afferent nerve fibers to primary afferent neurons (dorsal root ganglia) lead to sensitization and hyperexcitability of the dorsal root ganglia (DRG), which is rich in ion channels and sensitive to a variety of injurious signaling molecules, triggering a signaling cascade that continues to transmit nerve impulses and initiates the release of neurotransmitters in the anterior dorsal horn of the spinal cord such as growth inhibitors, calcitonin gene-related peptides, and substance P (Liem et al., 2016). This activates the release of bradykinin, histamine, and other mediators at the site of injury (Liem et al., 2016). This activates spinal cord neurons and releases glutamate, which binds to N-methyl-D-aspartic acid (NMDA) receptors resulting in increased neuronal excitability, i.e., central sensitization, as a result of the continued peripheral input to the DRG (Almeida et al., 2004). Ultimately, the spinal cord continues to transmit pain signals to higher centers in the brain producing nociception. Chronic exposure to injurious stimuli over a long period of time leads to permanent changes in neuronal structure and function, ultimately resulting in intractable, persistent neuropathic pain (Dinakar and Stillman, 2016) (Tables 1, 2).

**TABLE 1:** Mechanisms of sphingolipid metabolism-related molecules involved in pain onset.

Sphingolipid metabolism-related molecules	Effects	Reference
Ceramides	In a sciatic nerve ligation mouse model of neuropathic pain, ceramide biosynthetic enzyme expression was upregulated in the spinal cord, whereas injection of a ceramide inhibitor significantly attenuated nociceptive hypersensitivity in mice	Kobayashi et al. (2012)
	The sensitizing effect of NGF is mediated by the activation of the sphingolipid metabolism-related signaling pathway to release ceramide	Zhang et al. (2002)
	NGF may enhance peripheral sensitization by activating the p75 receptor and releasing ceramides, which act as downstream signaling molecules	Zhang and Nicol (2004)
	SAFit2 resulted in reduced expression of ceramide synthase in lumbar DRGs, and a lipidomic screen of DRG and spinal cord revealed that SAFit2 significantly restored C16 dihydroceramide levels and reduced TRPV1 activation with release of calcitonin gene-related peptide (CGRP)	Wedel et al. (2022)
	Ceramide is dependent on NF-ɤB and p38 kinases mediate neuronal sensitization	Matsuoka et al. (2019)
	The upregulation of NLRP2 vesicles and IL-1β expression after plantar injection of ceramide in mice	Matsuoka et al. (2019)
S1P	S1P-induced pain behaviors are significantly reduced in S1PR3 knockout mice	Camprubí-Robles et al. (2013)
	S1P has been shown to mediate peripheral sensitization and nociceptive sensitization, and this effect has been associated with induction of neutrophil infiltration	Baccei et al. (2013)
	In a mouse model of sickle cell disease (SCD), S1P receptor activation leads to significantly elevated IL-6 levels involved in severe chronic inflammation and tissue damage	Zhao et al. (2018)
	S1P is a downstream signal for ceramide-induced nociception and that S1P acts in a manner that binds to S1PR.	Doyle et al. (2011a)
	S1P-S1PR pathway may be involved in pain development through the nitrite oxidation pathway	Doyle et al. (2011b)
	S1PR2 overexpression increased the pain threshold	Li et al. (2019)
	S1P is involved in transmitting pain signals by regulating multiple ion channels	Basso and Altier (2017)
	S1P promotes glutamate secretion in hippocampal neurons	Kajimoto et al. (2023)
	Addition of S1P to rat hippocampal slices increased AMPA receptor-mediated postsynaptic currents	Kanno et al. (2010)
	Knockout of receptor S1PR3 in a mouse model affects hippocampal neuronal excitability	Weth-Malsch et al. (2016)
	In primary cultured rat astrocytes, S1P was shown to release the pro-inflammatory cytokine CXCL1 through activation of MAPK signaling via the TRPC6 channel leading to calcium inward currents	Shirakawa et al. (2017)
	In primary cultured rat astrocytes, S1P was shown to release the pro-inflammatory cytokine CXCL1 through activation of MAPK signaling via the TRPC6 channel leading to calcium inward currents	Shirakawa et al. (2017)
	Neuroinflammation induced by altered astrocyte phenotypes was an important mechanism by which the S1P-S1PR1 axis is involved in pain progression	Chen et al. (2019)
	High expression of S1PR3 in astrocytes induced upregulation of the inflammatory response	Dusaban et al. (2017)
SMase	Sphingomyelin enzymes have an effect on pain status primarily by regulating ceramide metabolism production	Ong et al. (2015)
Lipid raft	Lipid rafts in glial cells are localized tissue matrices for membrane receptors and channels involved in neuroinflammation and pain processing, and increased levels of inflammatory receptors, ligand molecules, ion channels, and enzymes in lipid rafts contribute to the activation of neuroglial cell phenotypic changes and inflammatory signaling	Miller et al. (2020b)
Ganglioside	Gangliosides are mainly involved in sensitization processes through participation in nerve injury, which is associated with pain	Lim et al. (2020)
	GT1b in the spinal cord was detected to be significantly upregulated in mice modeling peripheral nerve injury and involved in central sensitization and pain onset through activation of TLR2-induced microglia activation	Lim et al. (2020)
	Damaged neuron-derived GT1b is involved in central sensitization by activating microglia through the induction of pro-inflammatory mediator release	Lee et al. (2021)
	GM3 depletion reversed neuropathic changes and improved pain and wound healing status	Menichella et al. (2016)
	GM3 synthase knockout diabetic mice were observed to block hyperexcitability-associated calcium inward flow to diabetic dorsal root ganglion neurons, thereby completely reversing the resulting neuropathic pain	Menichella et al. (2016)

**TABLE 2:** The role of sphingolipid metabolism in diverse pain conditions.

Disease	Molecular targets	Effects
Morphine anti-injury tolerance	S1P	Continuous administration of morphine treatment activates metabolic mechanisms of S1P and S1PR1 signaling production in the CNS, leading to morphine tolerance
	Ceramide	Ceramide promotes morphine resistance to injury demonstrated that ceramide is an upstream signaling mediator of neuroimmune activation, and that inhibition of ceramide synthesis blocks morphine resistance to injury, a process that is achieved by intervening in nitrogen oxidative stress through peroxynitrite synthesis
	Ceramide	Ceramide also induces oxidative DNA damage and activation of the nuclear enzyme poly adenosine diphosphate-ribose polymerase (PARP) via the nitrite-mediated nitrogen oxidative stress pathway, leading to apoptosis and participation in tolerance generation
	S1P	S1P inhibitors have likewise been shown to block the development of morphine tolerance by improving neuroglial cell function and decreasing the associated proinflammatory cytokine production
	S1PR1	S1PR1 antagonists blocked the development of morphine tolerance and prevented morphine-induced neuropathic pain
Fabry disease (FD)	Sphingolipids	The accumulation of sphingolipids in the nervous system (mainly peripheral nerves and DRG) alters the morphology and function of neuronal cytosol and axon, and regulates the activity of ion channels, which leads to sensory abnormalities and chronic pain
	Gb3/Lyso-Gb3	Excess Gb3 and lyso-Gb3 in peripheral tissues have a direct sensitizing effect on neurons
	Lyso-Gb3	Lyso-Gb3 can be involved in sensitizing peripheral neurons by acting on voltage-dependent calcium channels
	Gb3	One study analysis confirmed that tumor necrosis factor gene expression was higher in FD patients than in controls, and it was also hypothesized that there is a feed-forward loop between tumor necrosis factor, Gb3, and FD-induced pain, in which tumor necrosis factor further stimulates Gb3 loading of neurons and alters the expression of pain-associated ion channels, thus contributing to the analgesic effect
	Gb3	Gb3 induces mechanical nociceptive hypersensitivity in mice by enhancing proNGF-p75NTR signaling
	Gb3	Higher Gb3 deposition in skin fibroblasts may promote the release of proinflammatory mediators through activation of the Notch1 signaling pathway, thereby creating a peripheral inflammatory environment that promotes sensitization of injury receptors
Cancer-related pain	S1P/S1PR1 axis	Paclitaxel-induced neuropathic pain is associated with activation of the S1P/S1PR1 axis
	S1P/S1PR1 axis	S1PR1 antagonists act by blocking spinal neuroinflammation through inhibition of the activation of NF-ɤB and MAPKs
	S1P/S1PR1 axis	Bortezomib treatment resulted in increased levels of ceramides, sphingomyelins, and S1P in DHSc, demonstrating that Bortezomib upregulates S1P by altering the ceramide metabolic pathway, leading to pain, and this process is accompanied by increases in tumor necrosis factor and IL-1β, as well as changes in glutamatergic synaptic activity
	S1P/S1PR1 axis	The S1P/S1PR1 axis mediates CIPN development by affecting the primary cellular substrate, astrocytes, and driving the corresponding neuroinflammation and changes in glutamatergic synaptic activity
	S1P/S1PR1 axis	Intrathecal and systemic administration of S1PR1 antagonists can reverse CIBP pain behavior
Multiple sclerosis (MS)	S1P	S1P has strong pro-inflammatory activity, and in a clinical study, elevated concentrations of S1P were detected in the cerebrospinal fluid of MS patients, suggesting that S1P is involved in MS-associated chronic inflammation
	S1P	FTY720 effectively ameliorated the symptoms of an experimental autoimmune encephalomyelitis (EAE) model. It was also revealed that its mechanism of action may include affecting lymphocyte trafficking and initial activation, thereby reducing spontaneous lymphocyte infiltration into inflammatory sites
	Ceramide/S1P	Disturbances in sphingolipid metabolism and abnormal accumulation of ceramide in astrocytes may be involved in the demyelination process by damaging oligodendrocytes

Spinal cord glial cells, primarily microglia and astrocytes, also serve an important role in the development and maintenance of pain, driving neuroinflammation to central sensitization (Ji et al., 2018). Microglia are the primary effector cells in the spinal cord, capable of detecting spinal cord neuronal injury and rapidly extending to the damaged area (Wake et al., 2013). Microglia undergo reactive hypertrophy and proliferation and release large amounts of inflammatory mediators and proinflammatory cytokines through inflammatory pathways such as Toll-like receptor 2 (TLR2/TLR4) activation, which excites neurons leading to sensitization (Inoue and Tsuda, 2018). Astrocytes fill in between neuronal cytosol and synapses, playing a trophic and supportive role (Ji et al., 2019). Nerve injury is accompanied by astrocyte proliferation and increased expression of glial fibrillary acidic protein (GFAP), forming a glial scar (Ji et al., 2013). Astrocytes are also involved in the development of pain, and a study reported that hypertrophy of astrocytes in the Spinal cord dorsal horn (SDH) was associated with pain hypersensitivity after peripheral nerve injury in rats. Moreover, transgenic expression of the pro-inflammatory cytokines by astrocytes increases mechanical nociceptive abnormalities in a mouse model of nerve injury, suggesting that astrocytes mediate pain through pro-inflammatory cytokines.

## 5 Mechanisms of sphingolipid metabolism-related molecules involved in pain onset

Sphingolipids are involved in neurodegeneration and a variety of inflammatory responses, whereas disturbances in sphingolipid metabolism have been shown to be strongly associated with a variety of neurologic pathologies (Czubowicz et al., 2019). S1P and its bioactive precursor ceramide mediate multiple injurious signaling cascade responses and inflammation (Czubowicz et al., 2019). In a metabolomics study using tibial nerve transection (TNT) rat specimens, metabolic alterations in the spinal cord are critical in maintaining neuropathic pain, whereas dysregulation of sphingolipid and ceramide metabolites induces mechanical nociceptive abnormalities as well as inducing release of associated cytokines from astrocytes (Patti et al., 2012).

### 5.1 Ceramides

Ceramides act as lipid mediators that modulate pain sensitivity (Malan and Porreca, 2005). In a sciatic nerve ligation mouse model of neuropathic pain, ceramide biosynthetic enzyme expression was upregulated in the spinal cord, whereas injection of a ceramide inhibitor significantly attenuated nociceptive hypersensitivity in mice (Kobayashi et al., 2012). Tumour necrosis factor α (TNF-α), an inflammatory mediator, acts on sensory neurons to induce neuropathic pain. Inflammatory diseases or peripheral nerve injuries can result in the release of large amounts of TNF-α, which can produce pain-related behaviors through the downstream second messenger ceramide (Joseph and Levine, 2004). TNFR stimulates the activation of SMase through TNFR binding thereby upregulating ceramide and promoting pain. Nerve growth factor (NGF) is one of the important peripheral mediators in the mechanism of nociceptive sensitization, binding to TrkA receptors and p75 receptors on the surface of neurons thereby enhancing excitability and sensitivity (Zhang and Nicol, 2004). Zhang et al.’s experiments revealed that the sensitizing effect of NGF is mediated by the activation of the sphingolipid metabolism-related signaling pathway to release ceramide and demonstrated that NGF may enhance peripheral sensitization by activating the p75 receptor and releasing ceramides, which act as downstream signaling molecules (Zhang et al., 2002; Zhang and Nicol, 2004). FK506-binding protein 51 (FKBP51) maintains long-term pain states by regulating glucocorticoid signaling, and its inhibitor SAFit2 significantly reduces mechanical hypersensitivity in the model (Maiarù et al., 2018). In a mouse model of SNI, SAFit2 resulted in reduced expression of ceramide synthase in lumbar DRGs, and a lipidomic screen of DRG and spinal cord revealed that SAFit2 significantly restored C16 dihydroceramide levels and reduced TRPV1 activation with release of calcitonin gene-related peptide (CGRP) (Wedel et al., 2022).

Patti et al. (Patti et al., 2012) observed significant upregulation of the levels of ceramide and its metabolites sphingosine and dimethylsphingosine (DMS) by mass spectrometry analysis of plasma and tissues from TNI mice. DMS activates astrocytes, increases intracellular calcium ion concentration, inhibits glutamate uptake and increases spillover, which leads to over-activation of NMDA receptors and induces neuropathic rational pain (Nie and Weng, 2010; Mühle et al., 2013). In addition, to clarify the specific signaling pathways involved in neuronal sensitization by ceramide, Doyle et al. injected ceramide into the rat plantar foot and observed the development of mechanical pain sensitization and a significant increase in cyclooxygenase-2 (COX-2) and Prostaglandin E2 (PGE2) in rats, and further demonstrated that this effect of ceramide is dependent on NF-ɤB and p38 kinase mediation (Matsuoka et al., 2019). A recent study observed upregulation of NLRP2 vesicles and IL-1β expression after plantar injection of ceramide in mice, confirming the involvement of NLRP2 inflammatory vesicles in ceramide-induced hypersensitivity (Matsuoka et al., 2019). Overall, ceramides play a potential role in peripheral sensitization and mechanical nociceptive sensitization through links with inflammation-related signals, which are inextricably linked to the onset and maintenance of pain.

### 5.2 S1P

S1P, a ceramide metabolite that also exerts potent inflammatory effects, exerts its biological effects mainly by being transported outside the cell to bind to G protein-coupled receptors, which are coupled to various G proteins (Spiegel and Milstien, 2003). Intracellular levels of S1P are tightly regulated by synthesis and degradation, and metabolic dysregulation is closely associated with the induction of inflammation (Takabe et al., 2008). There are five identified S1P receptors (S1PR1-5), which are widely distributed in the nervous system, among which sensory neurons mainly express S1PR1 and S1PR2, DRG mainly expresses S1PR3, and the CNS is richly expressed in all except S1PR4 (Hashimoto et al., 2011; Camprubí-Robles et al., 2013). S1P and S1PR are thought to be key factors in the regulation of receptor excitability (Hill et al., 2018). S1P-induced pain behaviors are significantly reduced in S1PR3 knockout mice (Camprubí-Robles et al., 2013). And it has been demonstrated that this is achieved by regulating KCNQ2/3 channels and blocking the current (Hill et al., 2018).

S1P has been shown to mediate peripheral sensitization and nociceptive sensitization, and this effect has been associated with induction of neutrophil infiltration (Baccei et al., 2013). In a mouse model of sickle cell disease (SCD), S1P receptor activation leads to significantly elevated IL-6 levels involved in severe chronic inflammation and tissue damage (Zhao et al., 2018). S1P is also thought to be released from immune cells during injury and to be involved in hypersensitivity by activating sensory neuron excitability through linkage to G proteins (Jolly et al., 2004; Zhang et al., 2006). Ceramides have been shown to induce peripheral sensitization, and the administration of inhibitors of SphK1 and SphK2 and an S1PR1 antagonist, respectively, in a rat model of plantar injection of ceramides, found that they both reduced nociceptive sensitization, demonstrating that S1P is a downstream signal for ceramide-induced nociception and that S1P acts in a manner that binds to S1PR(Doyle et al., 2011a). Direct plantar injection of S1P in rats also induced thermal nociception, and S1PR1 receptor antagonists blocked this effect in a dose-dependent manner, again demonstrating that S1P acts through the S1P-S1PR pathway (Doyle et al., 2011b). Meanwhile, Doyle et al. further demonstrated that the S1P-S1PR pathway may be involved in pain development through the nitrite oxidation pathway (Doyle et al., 2011b). In an animal model of chronic compression injury (CCI), the expression of S1PR2 mRNA, another receptor for S1P, was found to decrease and then an increase at 14 days, and S1PR2 overexpression increased the pain threshold, while elevated levels of inflammatory factors, such as IL-1β, I L-6, and CCl-2, were found (Li et al., 2019).

Notably, multiple ion channels in peripheral neurons are regulated by S1P. S1P activates ligand-gated ion channels by coupling to G proteins, including transient receptor potential anchor protein 1 (TRPA1), canonical transient receptor potential (TRPC), and transient receptor potential vanilloid 1 (TRPV1) (Hashimoto et al., 2011; Squillace et al., 2020; Sun et al., 2020). The above are widely expressed on injury receptors and are involved in transmitting pain signals (Basso and Altier, 2017). In addition, S1P can stimulate the triggering of two voltage-gated chloride channels (CLCN), CLCN3 and CLCN5, to generate excitatory currents (Qi et al., 2018).

Peripheral persistent injurious signals stimulate injurious receptors, which through signaling lead to increased release of neurotransmitters (mainly glutamate) from the dorsal horn of the spinal cord, which bind to receptors such as NMDA and AMPA leading to changes in CNS neuronal plasticity and hyperexcitability and activation, and ultimately central sensitization to maintain the pain state (Ji et al., 2018). Kajimoto et al. demonstrated that exogenous S1P promotes glutamate secretion in hippocampal neurons (Kajimoto et al., 2023). Addition of S1P to rat hippocampal slices increased AMPA receptor-mediated postsynaptic currents, demonstrating its role in synaptic excitatory transmission (Kanno et al., 2010). Knockout of receptor S1PR3 in a mouse model affects hippocampal neuronal excitability (Weth-Malsch et al., 2016).

Central sensitization is simultaneously driven by neuroinflammation in the CNS, which results from the activation of neuroglia that exert an immune effect to release inflammatory signals (Ji et al., 2018). In primary cultured rat astrocytes, S1P was shown to release the pro-inflammatory cytokine CXCL1 through activation of MAPK signaling via the TRPC6 channel leading to calcium inward currents (Shirakawa et al., 2017). In a recent study, after intrathecal injection of the S1PR1 agonist, SEW2871, into mice, the production of mechanical nociceptive sensitization was observed and NLRP3 was detected activation and IL-1b production, demonstrating that S1P drives neuroinflammation in the dorsal horn of the spinal cord through activation of downstream NLRP3 and NLRP3 signaling, which subsequently induces pain (Doyle et al., 2019). At the same time, this study linked the above neuroinflammatory response to astrocytes, and the absence of mechanical nociceptive hypersensitivity after knocking out the astrocyte-specific S1PR1 gene in mice further demonstrated that the key cellular locus is precisely S1PR1 in astrocytes (Doyle et al., 2019). Chen et al. similarly demonstrated that neuroinflammation induced by altered astrocyte phenotypes was an important mechanism by which the S1P-S1PR1 axis is involved in pain progression (Chen et al., 2019). In an *in vitro* experiment, high expression of S1PR3 in astrocytes induced upregulation of the inflammatory response (Dusaban et al., 2017). As for microglia, which are also involved in driving constitutive neuroinflammation, it has been found that the addition of exogenous S1P enhances their inflammatory response (Nayak et al., 2010).

### 5.3 Other molecules

Endogenous sphingolipids induce glial cell activation, proinflammatory mediator release, and nociceptive hypersensitivity (Wei et al., 2021). Pan et al. analyzed serum samples from patients with multisite musculoskeletal pain (MSMP) by metabolomics and found specific expression of several sphingolipids, suggesting that sphingolipids are involved in the associated pain (Pan et al., 2021). In a mouse model of “early life pain” (ELP), Vogel et al. revealed persistent peripheral sensitization from a calcium imaging perspective and found from brain and plasma lipid studies that this was associated with changes in the dynamic balance of sphingolipid metabolism in which sphingomyelinase may play an important role (Vogel et al., 2023). Sphingomyelin enzymes have an effect on pain status primarily by regulating ceramide metabolism production (Ong et al., 2015). Administration of an acid sphingomyelinase (aSMase) inhibitor is effective in reducing mechanical anomalous pain induced by facial carrageenan injections (Ong et al., 2015). The IL-1β receptor-dependent neutral sphingomyelinase/ceramide signaling pathway mediates functional coupling with presynaptic NMDA receptors (Yan and Weng, 2013). In addition, the IL-1β receptor-dependent neutral sphingomyelinase/ceramide signaling pathway mediates functional coupling with presynaptic NMDA receptors (Yan and Weng, 2013). In addition, aSMase expression and ceramide production are also significantly increased in the gray region around the aqueduct in mice with chronic morphine pellet implantation, whereas selective silencing of the aSMase gene by local aSMase shRNA transfection reduces the analgesic response to acute morphine, and aSMase activation and ceramide production play a major role in morphine tolerance (Ritter et al., 2012).

In cell membranes, dynamic aggregation of sphingolipids and cholesterol can form lipid rafts within the bilayer that are involved in attachment of proteins and signaling (Simons and Ikonen, 1997). Lipid rafts in glial cells are localized tissue matrices for membrane receptors and channels involved in neuroinflammation and pain processing, and increased levels of inflammatory receptors, ligand molecules, ion channels, and enzymes in lipid rafts contribute to the activation of neuroglial cell phenotypic changes and inflammatory signaling (Miller et al., 2020b). For example, lipid rafts can modulate ion channel opening by interacting with TRP channels (Sághy et al., 2015). Also, cleavage of sphingomyelin by SMase, when given, inhibits the release of CGRP from sensory nerve endings and reduces TRP activation-associated nociceptive sensitization, demonstrating the role of the structural integrity of lipid rafts in the maintenance of pain states, and that targeting this structure could be a potential alternative to pain medications (Sághy et al., 2015; Horváth et al., 2021). A study by Woller et al. used Apolipoprotein A-I Binding Protein (AIBP) to reduce lipid raft abundance by removing cholesterol, thereby reversing aberrant pain induced by neuroinflammation in mice (Woller et al., 2018).

Gangliosides are sialylated sphingolipids that are highly expressed in the nervous system and are also involved in the composition of cell membrane lipid rafts. Neurons are enriched in gangliosides, which include four main types: GM1, GD1a, GD1b and GT1b. Notably, gangliosides are highly plastic, with significant differences in types in different neurological disorders, and are mainly involved in sensitization processes through participation in nerve injury, which is associated with pain (Lim et al., 2020). GT1b in the spinal cord was detected to be significantly upregulated in mice modeling peripheral nerve injury and involved in central sensitization and pain onset through activation of TLR2-induced microglia activation (Lim et al., 2020). Ganglioside synthase knockout mice exhibit spinal cord inflammation and neurodegenerative lesions with increasing age and are observed to have aggregation of microglia and aberrant proliferation of astrocytes as well as increased expression of inflammatory cytokines. Lee et al. also showed that damaged neuron-derived GT1b is involved in central sensitization by activating microglia through the induction of pro-inflammatory mediator release (Lee et al., 2021). And, in diabetic small fiber neuropathy and secondary neuropathic pain, GM3 depletion reversed neuropathic changes and improved pain and wound healing status. Isolation of the DRG and sciatic nerve in diabetic mice resulted in a significant increase in GM3 expression levels compared to controls (Menichella et al., 2016). GM3 synthase knockout diabetic mice were observed to block hyperexcitability-associated calcium inward flow to diabetic dorsal root ganglion neurons, thereby completely reversing the resulting neuropathic pain (Menichella et al., 2016). It also improves glucose tolerance in mice, indirectly ameliorates small fiber lesions, and is neuroprotective (Menichella et al., 2016).

## 6 The role of sphingolipid metabolism in diverse pain conditions

### 6.1 Morphine anti-injury tolerance

Morphine is a potent and effective analgesic for pain relief, but the development of tolerance after long-term administration is one of the major challenges facing pain management. Previous studies have revealed part of the mechanism of injury-resistant tolerance to morphine, which is associated with neuroimmune activation leading to proinflammatory cytokine release, overproduction of reactive oxygen and nitrogen species in the spinal cord, and neuronal apoptosis (Mayer et al., 1999; Watkins et al., 2007; Grace et al., 2015). Continuous administration of morphine treatment activates metabolic mechanisms of S1P and S1PR1 signaling production in the CNS, leading to morphine tolerance (Salvemini and Doyle, 2023). Nitrite is a potent pro-inflammatory nitrogen oxidizer, and Muscoli et al. further demonstrated the involvement of nitrite in the spinal cord in anti-injury tolerance through the molecular mechanisms of induced neuroimmune activation and the release of factors such as TNF-α, IL-1β, and pro-apoptotic effects (Muscoli et al., 2007). Whereas ceramide serves as a potent pro-inflammatory and pro-apoptotic signature molecule, a recent study addressing the mechanism by which ceramide promotes morphine resistance to injury demonstrated that ceramide is an upstream signaling mediator of neuroimmune activation, and that inhibition of ceramide synthesis blocks morphine resistance to injury, a process that is achieved by intervening in nitrogen oxidative stress through peroxynitrite synthesis (Ndengele et al., 2009). Subsequently, Bryant et al. demonstrated that ceramide also induces oxidative DNA damage and activation of the nuclear enzyme poly adenosine diphosphate-ribose polymerase (PARP) via the nitrite-mediated nitrogen oxidative stress pathway, leading to apoptosis and participation in tolerance generation (Bryant et al., 2009). In addition, S1P inhibitors have likewise been shown to block the development of morphine tolerance by improving neuroglial cell function and decreasing the associated proinflammatory cytokine production (Muscoli et al., 2010; Doyle et al., 2020a). In a mouse model of neuropathic pain, S1PR1 antagonists blocked the development of morphine tolerance and prevented morphine-induced neuropathic pain by reversing S1P-induced neuroinflammation including activation of mitogen-activated protein kinase p38 and NF-κB, and increased expression of inflammatory cytokines (Doyle et al., 2020b). This reveals that S1P is involved in morphine anti-injury tolerance by activating S1PR1 and affecting downstream inflammatory signaling.

### 6.2 Fabry disease (FD)

Fabry disease is an inherited disorder caused by mutations in the gene encoding expression of α-galactosidase A (α-GalA), and one of its typical clinical symptoms is severe neuropathic pain, including severe paroxysmal pain, hypersensitivity to mechanical stimuli, and chronic pain (Bernardes et al., 2020). α-GalA is a lysosomal hydrolase that catalyzes the removal of terminal α-galactose residues from glycosylated molecules. His absence, however, affects the degradation and recirculation of major intracellular substances, resulting in the accumulation of glycosylation products such as globotriosylceramide (Gb3), lysotriosylceramide (lyso-Gb3), and bis-galactosylceramide in the lysosome (Miller et al., 2020a). Of these, globotriosylsphingosine (lyso-Gb3) is a deacylated form of globotriosylceramide (Gb3). The accumulation of sphingolipids in the nervous system (mainly peripheral nerves and DRG) alters the morphology and function of neuronal cytosol and axon, and regulates the activity of ion channels, which leads to sensory abnormalities and chronic pain (Burand and Stucky, 2021). Differential expression of mRNAs related to “ceramide metabolism” was determined by expression profiling in the DRG of Fabry mice (Kummer et al., 2018).

Choi et al. found that direct injection of Gb3 or lyso-Gb3 into the paws of mice induced a Fabry-like phenotype and caused mechanically abnormal pain, implying that excess Gb3 and lyso-Gb3 in peripheral tissues have a direct sensitizing effect on neurons (Choi et al., 2015). Gb3 deposition in DRG neurons observed in α-GAL-deficient mice (GALKO) correlates with increased TRPV1 protein associated with TRPV1 channels providing evidence for a potential mechanism of Gb3-induced pain (Hofmann et al., 2018). Activation of voltage-gated sodium channels has likewise been shown to be a possible electrophysiological mechanism for Gb3 action on injury receptors (Glorioso et al., 2014; Namer et al., 2017). Isolated cultured mouse DRG neurons were given exogenous lyso-Gb3 and a concentration-dependent increase in calcium ion levels as well as an increase in the current density of voltage-dependent calcium channels in small-diameter dorsal root ganglion neurons were observed, demonstrating that lyso-Gb3 can be involved in sensitizing peripheral neurons by acting on voltage-dependent calcium channels (Choi et al., 2015). One study analysis confirmed that tumor necrosis factor gene expression was higher in FD patients than in controls, and it was also hypothesized that there is a feed-forward loop between tumor necrosis factor, Gb3, and FD-induced pain, in which tumor necrosis factor further stimulates Gb3 loading of neurons and alters the expression of pain-associated ion channels, thus contributing to the analgesic effect (Üçeyler et al., 2019). Similarly, NGF, a key molecule that plays a role in neuropathic pain, has been focused on mechanistic studies of Gb3-induced pain. Sugimoto et al. found that blockade of proNGF and p75NTR, but not mNGF and TrkA, attenuated Gb3-induced mechanical hyperalgesia (Sugimoto et al., 2021). And Gb3 injection did not alter the expression levels of NGF and its receptors, suggesting that Gb3 induces mechanical nociceptive hypersensitivity in mice by enhancing proNGF-p75NTR signaling (Sugimoto et al., 2021). In addition, a study of skin puncture biopsies from FD patients and controls confirmed that higher Gb3 deposition in skin fibroblasts may promote the release of proinflammatory mediators through activation of the Notch1 signaling pathway, thereby creating a peripheral inflammatory environment that promotes sensitization of injury receptors (Rickert et al., 2020). The evidence from the above studies amply demonstrates that impaired metabolism of sphingolipids, especially ceramides, is an important link in the development of FD pain.

### 6.3 Cancer-related pain

More than half of patients with advanced cancer suffer from long-term chronic pain, and opioids do not give them the efficacy they expect (Magee et al., 2019). Cancer-related pain is divided into several major patterns. For example, direct tumor compression as well as invasion of tumor tissue can lead to local nerve injury, release of inflammation-related mediators leading to neuropathic pain (Xu et al., 2022). Bone metastases can lead to an imbalance in the role between osteoblasts and osteoclasts, causing changes in the bone microenvironment and inducing peripheral and central sensitization, which can lead to secondary inflammation as well as neuropathic pain (Xu et al., 2022). In addition, a category of cancer-related pain that should be of particular interest and study is pain secondary to chemotherapeutic agents. Specifically, chemotherapeutic agents such as paclitaxel and platinum-containing agents are potent neurotoxic agents that cause pain from peripheral neuropathy (Xu et al., 2022). The primary target of oxaliplatin neurotoxicity is the DRG. Specific mechanisms by which chemotherapeutic agents cause pain that have now been revealed involve increased peripheral proinflammatory cytokines, mitochondrial dysfunction, transient receptor potential channels, and ion channels (Brandolini et al., 2019; Colvin, 2019).

S1P and SphK1 have been shown to be closely related to tumor metastasis and progression, and SphK1 signaling can promote tumor metastasis and resist apoptosis. Enhanced S1PR1 signaling has also been shown to consistently activate NF-κB and STAT3, the major transcription factors associated with tumor growth and metastasis (Deng et al., 2012; Nagahashi et al., 2014; Acharya et al., 2019). The S1PR1 antagonist FTY720 can enhance the efficacy of chemotherapeutic agents by inhibiting S1P signaling. The S1P signaling enhances the efficacy of chemotherapeutic agents, and its combination with low-dose adriamycin has been shown to synergistically inhibit the progression of triple-negative breast cancer (Katsuta et al., 2017). In contrast, ceramides play an important role in antitumor therapy. Many anticancer drugs have been shown to increase endogenous ceramide levels and modulate downstream protein targets and signaling pathways, thereby inducing apoptosis in tumor cells (Ogretmen and Hannun, 2004). It has been shown that paclitaxel-induced neuropathic pain is associated with activation of the S1P/S1PR1 axis (Janes et al., 2014). In a rat model of paclitaxel-induced mechanical nociceptive hypersensitivity, increased formation of S1P and SphK1 in the dorsal horn of the spinal cord was detected, whereas administration of an S1PR1 antagonist blocked the progression of paclitaxel-induced pain (Janes et al., 2014). Also, this study further demonstrates that S1PR1 antagonists act by blocking spinal neuroinflammation through inhibition of the activation of NF-ɤB and MAPKs (Janes et al., 2014). In another study, after administration of systemic docetaxel to mice, the levels of 1-deoxy SL in plasma and DRG were upregulated, which is a specific indicator of chemotherapeutic agent-induced neuropathy, demonstrating a correlation between dysregulation of sphingolipid metabolism and chemotherapeutic agent-induced neuropathic pain (Kramer et al., 2015; Becker et al., 2020). Bortezomib, which is used for the treatment of multiple myeloma and non-Hodgkin’s lymphoma, is also often accompanied by chemotherapy-induced peripheral neuropathy (CIPN) during its use. Stockstill et al. analyzed this by LC-ESI-MS/MS cascade technique and found that Bortezomib treatment resulted in increased levels of ceramides, sphingomyelins, and S1P in DHSc, demonstrating that Bortezomib upregulates S1P by altering the ceramide metabolic pathway, leading to pain, and this process is accompanied by increases in tumor necrosis factor and IL-1β, as well as changes in glutamatergic synaptic activity (Stockstill et al., 2018). The specific mechanism of action is thought to be related to astrocytes, and mice with astrocyte-specific S1PR1 deletions did not develop neuropathic pain, suggesting that the S1P/S1PR1 axis mediates CIPN development by affecting the primary cellular substrate, astrocytes, and driving the corresponding neuroinflammation and changes in glutamatergic synaptic activity (Stockstill et al., 2018).

As for tumor-induced bone cancer pain (CIBP), Grenald et al. addressed the relationship between the S1P/S1PR1 axis and bone cancer pain. In a mouse model of breast cancer bone metastasis, ceramide levels were significantly decreased and the ceramide degradation product, S1P, was increased compared to controls (Grenald et al., 2017). Reversal of CIBP pain behavior after intrathecal and systemic administration of S1PR1 antagonists demonstrates the role of dysregulated sphingolipid metabolism and the S1P/S1PR1 axis in CIBP (Grenald et al., 2017).

In summary, abnormal sphingolipid metabolism plays an important role in tumor metastasis and progression, and for tumor-associated pain, the S1P/S1PR1 axis seems to play a unique role in inducing neuropathic pain mainly by affecting astrocytes to mediate glial cell-neuron interactions that drive neuroinflammation and sensitization both centrally and peripherally. S1PR1 antagonists in alleviating this type of pain with significant advantages.

### 6.4 Multiple sclerosis (MS)

Multiple sclerosis is an autoimmune-induced demyelinating lesion of the central nervous system, marked by multifocal destruction of myelin sheaths, mainly by macrophages and T-cells infiltrating into the central nervous system and myelin degradation. Axons lose the protection and support of myelin, resulting in neurologic deficits and sensory abnormalities due to increased sensitivity to external stimuli (Urits et al., 2019). The first symptom of MS is central neuropathic pain, which is primarily due to demyelinating lesions in relevant areas of the CNS (Solaro et al., 2012). Myelin sheaths are uniformly thick membranes characterized by multilayered stacking, with the main constituents being proteins and lipids, of which sphingolipids are the major lipid component, and the density of lipid stacking affects sphingolipid structure and function (Stadelmann et al., 2019). The protrusions of oligodendrocytes are connected to myelin sheaths, which form myelin sheath wrappings around axons to stabilize nerve conduction and metabolic support (Simons and Nave, 2016; Stadelmann et al., 2019).

Oligodendrocyte damage plays a role in demyelinating lesions in MS. The effects of sphingolipids on myelin are not only reflected in their involvement in the composition of the basic structure of myelin in the central nervous system, but also as bioactive modulators that influence glial cell-related signaling and thus regulate myelin formation (Podbielska et al., 2022). S1P has strong pro-inflammatory activity, and in a clinical study, elevated concentrations of S1P were detected in the cerebrospinal fluid of MS patients, suggesting that S1P is involved in MS-associated chronic inflammation (Kułakowska et al., 2010). Using S1P as a drug target for the treatment of MS, FTY720 has been approved by the FDA as the first oral therapy for the treatment of relapsing multiple sclerosis. FTY720 effectively ameliorated the symptoms of an experimental autoimmune encephalomyelitis (EAE) model (Foster et al., 2007). It was also revealed that its mechanism of action may include affecting lymphocyte trafficking and initial activation, thereby reducing spontaneous lymphocyte infiltration into inflammatory sites (Matloubian et al., 2004). FTY720 also affects T cell differentiation and function, reducing autoimmune T cells (Foster et al., 2007; Podbielska et al., 2012). The relative balance of ceramide and S1P as important regulators determines cell fate. In a co-culture system of neurons and oligodendrocytes, astrocystin-induced cell death was found to be associated with increased ceramide levels as well as decreased S1P levels (Qin et al., 2010). This study simultaneously detected a decrease in S1P levels in MS plaques (Qin et al., 2010). A study by Kim et al. revealed that disturbances in sphingolipid metabolism and abnormal accumulation of ceramide in astrocytes may be involved in the demyelination process by damaging oligodendrocytes (Kim et al., 2011). Thus, sphingolipids and sphingolipid metabolism play an important role in the progression of MS, with ceramides playing a major role along with S1P, which mediates neuroinflammation by affecting lymphocyte trafficking and immune activation, and ceramides inducing damage and apoptosis in oligodendrocytes. Targeted modulation of sphingolipid metabolism, especially the ceramide-S1P metabolic pathway, has great potential in promoting myelin repair as well as MS therapy.

## 7 Conclusion

Sphingolipids, as an important component of the plasma membrane of eukaryotic cells, can form lipid rafts within the bilayer, which are involved in the attachment of proteins and signal transduction. The various products of its metabolic pathway can also participate in intracellular signaling as second messengers and affect cellular activities such as proliferation, differentiation, and apoptosis. Many studies have shown a high correlation between sphingolipids and pain through metabolomic analysis. Recently, several studies have revealed the involvement of sphingolipids and sphingolipid metabolism in the development of pain, especially the core molecules of sphingolipid metabolism, ceramides, and the downstream S1P, and targeting these molecules can alleviate pain. The specific mechanisms involved in pain induction may be related to neuroinflammation. Or they act with neuroimmune cells such as astrocytes to induce the formation of an inflammatory microenvironment and promote peripheral and central sensitization. Targeting sphingolipid metabolism-related molecules such as ceramide or S1P has been validated to alleviate mechanical nociceptive sensitization in model mice, and the S1P receptor agonist, FTY720, as a drug approved for clinical use has a significant role in pain relief, and the future development of drugs targeting ceramides or the S1P/S1PR axis-related drugs is crucial for exploring the treatment of pain.
